# Identification of evolutionarily conserved DNA damage response genes that alter sensitivity to cisplatin

**DOI:** 10.18632/oncotarget.13353

**Published:** 2016-11-15

**Authors:** Anna V. Gaponova, Alexander Y. Deneka, Tim N. Beck, Hanqing Liu, Gregory Andrianov, Anna S. Nikonova, Emmanuelle Nicolas, Margret B. Einarson, Erica A. Golemis, Ilya G. Serebriiskii

**Affiliations:** ^1^ Molecular Therapeutics, Fox Chase Cancer Center, Philadelphia, PA 19111, USA; ^2^ Department of Biochemistry and Biotechnology, Kazan Federal University, Kazan 420008, Russian Federation; ^3^ Department of Biochemistry & Molecular Biology, Program in Molecular and Cell Biology and Genetics, Drexel University College of Medicine, Philadelphia, PA 19129, USA; ^4^ Department of Pharmaceutics, Jiangsu University, School of Pharmacy, Jingkou District Zhenjiang, Jiangsu 212013, China

**Keywords:** platinating agents, resistance, DNA damage response, head and neck cancer

## Abstract

Ovarian, head and neck, and other cancers are commonly treated with cisplatin and other DNA damaging cytotoxic agents. Altered DNA damage response (DDR) contributes to resistance of these tumors to chemotherapies, some targeted therapies, and radiation. DDR involves multiple protein complexes and signaling pathways, some of which are evolutionarily ancient and involve protein orthologs conserved from yeast to humans. To identify new regulators of cisplatin-resistance in human tumors, we integrated high throughput and curated datasets describing yeast genes that regulate sensitivity to cisplatin and/or ionizing radiation. Next, we clustered highly validated genes based on chemogenomic profiling, and then mapped orthologs of these genes in expanded genomic networks for multiple metazoans, including humans. This approach identified an enriched candidate set of genes involved in the regulation of resistance to radiation and/or cisplatin in humans. Direct functional assessment of selected candidate genes using RNA interference confirmed their activity in influencing cisplatin resistance, degree of γH2AX focus formation and ATR phosphorylation, in ovarian and head and neck cancer cell lines, suggesting impaired DDR signaling as the driving mechanism. This work enlarges the set of genes that may contribute to chemotherapy resistance and provides a new contextual resource for interpreting next generation sequencing (NGS) genomic profiling of tumors.

## INTRODUCTION

Platinating compounds including cisplatin, oxaliplatin, and carboplatin are mainstays of therapy for many cancers, including among others head and neck, ovarian, bladder, colorectal, and lung tumors [1]. These agents function primarily by modifying DNA, forming intrastrand crosslinks and other DNA lesions that, if unrepaired, lead to activation of cell death pathways in replicating cells; in additional secondary functions, interaction of platins with cytoplasmic targets increases oxidative stress and thereby provides an independent trigger of cell death [2]. When platins are applied at lower concentrations, cells undergo a transient arrest in the S and G2 phases of the cell cycle. This pause allows proteins in the nucleotide excision repair (NER) and homologous recombination (HR) pathways to eliminate platinum adducts and restore DNA integrity, preventing acquisition of deleterious mutations and abnormal mitoses. Beyond a lethal dosage threshold, activation of the ATM, ATR, and CHEK1 kinases initiates a signaling cascade that culminates in mitochondrial permeabilization and apoptosis [3–5].

Although platinum agents provide significant clinical benefit, tumors often develop resistance. Numerous mechanisms of resistance to platins have been described [6]. These include changes in the expression and activity of membrane transporters and the endocytic machinery, which reduce the intracellular concentration of platinating compounds; changes in heat shock proteins and other mediators of cellular stress response; changes in chromatin and DNA conformations that result in differential accessibility of the DNA target; transcription of genes that promote survival signaling; and changes in the DNA damage response (DDR) pathways that repair platinum and radiation induced DNA lesions [7]. These last mechanisms are of particular clinical interest, because they are associated with development of cross-resistance to multiple DNA damaging therapies, and therefore broadly reduce therapeutic options [8]. For example, overexpression of nucleotide excision repair (NER) proteins such as ERCC1 [9], proteins involved in translesion synthesis (also known as replicative bypass), including the polymerases POLH or REV3L [10], and proteins that mediate homologous recombination (HR) such as BRCA1 and BRCA2 [11–13], have all been associated with cisplatin resistance.

As genomic and proteomic data have become available in the past decade, it has become increasingly apparent that the number of proteins contributing to DDR processes is much greater than previously thought [14, 15]. Indeed a growing number of components involved in DNA repair systems have been defined. Based on analysis of these components, it has become clear that for many vital cellular processes, changes in gene expression or function that affect phenotypes of interest can be dispersed throughout extended signaling networks [16–18]. In this context, even as genomic profiling has become more standard in the clinic [19, 20], it remains a challenge to identify functionally essential components of the DDR response apparatus relevant to clinical resistance to cisplatin and other DNA damaging therapies.

We hypothesized that DDR genes are likely evolutionarily conserved, considering that maintenance of DNA integrity is critical for survival, which suggested a new approach to identify functionally important regulators of cisplatin resistance in human tumors. Based on this hypothesis, we analyzed data from the *Saccharomyces cerevisiae* genome database (SGD) and a large number of other functional screens for genes conferring of cisplatin resistance in lower organisms. To enrich the resulting dataset for genes relevant to DDR, we then integrated this information with additional data describing genes functionally defined as important for different classes of DDR, including data for *γ*-ray-, X-ray-, and UV-induced damage. Lastly, we directly tested human orthologs of genes identified in this manner for cellular responses to cisplatin in human cancer models, resulting in the identification of novel resistance mediators.

## RESULTS

### Integration of function-based datasets to define genes that regulate resistance to DNA damage in *S. cerevisiae*

**Figure 1:**
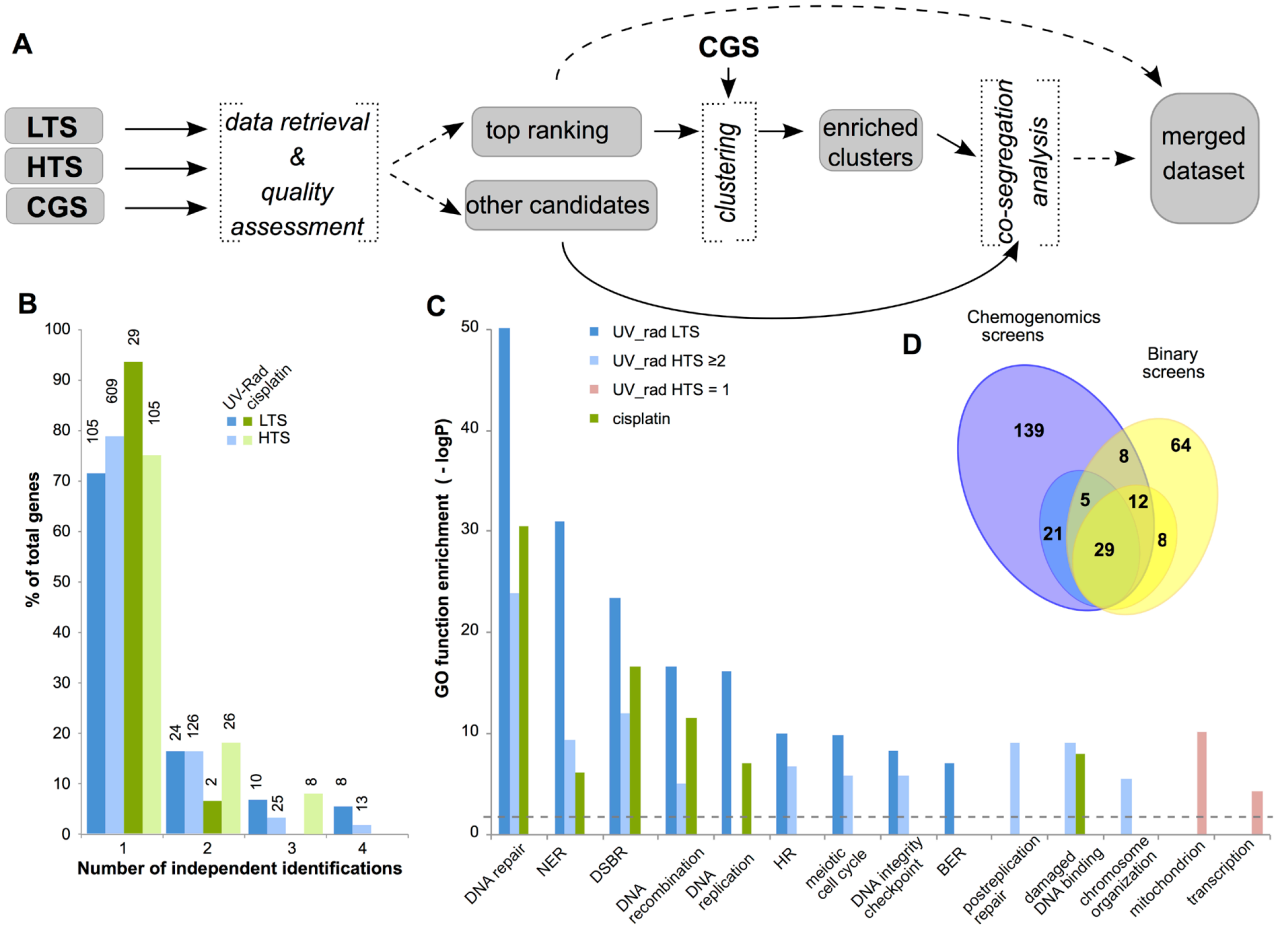
Identification of yeast genes mediating response to UV, X-ray radiation, or cisplatin **A**. Workflow for identification of yeast genes modulating sensitivity to UV, ionizing radiation, and cisplatin treatment. LTS, low throughput screen; HTS, high throughput screen; CGS, chemogenomic screen. **B**. Classification of candidate genes based on the number of independent studies that identified each gene as contributory to resistance to UV or radiation (UV_rad) or to cisplatin, as well as by type of supporting study (LTS and HTS). Y-axis indicates percent of genes for each class, while the absolute number of supporting studies is shown on the top of each bar. **C**. Enrichment in GO terms for specific subsets of candidate genes. NER, nucleotide excision repair; DSBR, double strand break repair; HR, homologous recombination; BER, base excision repair. Dashed line indicates the threshold for the statistical significance. **D**. Overlap between cisplatin resistance gene sets identified in binary LTS/HTS (yellow) and chemogenomic (blue) screens: numbers represent individual genes. Darker colors indicate genes supported by ≥2 binary screens, or by 3 independent chemogenomic studies; lighter colors indicate genes supported by 1 binary screen, or by 2 of 3 chemogenomic studies.

To test the hypothesis that DNA damage response genes in lower eukaryotes could identify human genes regulating cisplatin resistance, we extracted a first dataset from the SGD [21] based on phenotype-based query to identify genes where loss-of-function mutations altered sensitivity to *γ*-ray, X-ray, or UV irradiation and/or cisplatin treatment (832 and 126 genes, respectively; Figure 1A, 1B). This captured data from 105 low throughput screening (LTS) studies, typically characterizing 1-10 genes in detail. A second dataset was manually extracted from 12 published high throughput screening (HTS; defined as screening >15% of the genome) genetic studies [22–33], which was integrated with additional information from the SGD. For these datasets, results were reported in binary terms (sensitizing or not sensitizing).

Integrated analysis of genes designated as contributing to UV- and radiation-resistance from datasets 1 and 2 (designated LTS and HTS, subsequently) indicated that 29% and 21% of genes in these sets, respectively, were identified in two or more studies (Figure 1B). Genes identified in LTS were more likely to be repeatedly identified and demonstrated statistically significant enrichment of gene ontology (GO) terms associated with DNA repair, cell cycle and chromosomal organization (Figure 1C). Candidates identified by a single HTS (HTS=1) showed little or no enrichment in these gene ontology (GO) terms; however, a group of 164 candidates nominated by at least 2 studies (HTS≥2) demonstrated enrichment in gene ontology terms similar to the LTS subset (Figure 1C). Combined, the LTS and HTS≥2 groups identified 263 genes involved in *γ*-ray-, X-ray-, and UV-resistance (Supplementary Table 1). A similar analysis for genes specifically implicated in cisplatin-resistance yielded a total of 126 genes, with data from both LTS and HTS. The combined LTS/HTS dataset was highly enriched for gene ontology annotations associated with DNA repair, cell cycle and chromosomal organization (Figure 1C, Supplementary Table 2). This is consistent with the idea that the selection criteria employed were appropriate for identification of genes with a plausible, direct connection to regulation of cisplatin sensitivity.

To augment this analysis, an additional dataset was collected from a group of three functional chemogenomics screening studies (CGS) (http://fitdb.stanford.edu; [34, 35]). In these large studies, a genome-wide panel of yeast strains, each mutated in a single gene, was challenged with different drugs over broad concentration ranges, providing continuous measurements to characterize the relative importance of each of the mutated genes in terms of resistance. Genes identified in the binary screens (datasets 1 and 2) were more frequently found among genes with greater importance for drug resistance (higher “fitscores”) in the chemogenomics screens performed with cisplatin (Supplementary Figure 1). Using data extraction cutoffs selected to reduce the fraction of false positives (see Supplementary Materials and Methods), we considered genes identified in two or more chemogenomics studies as most important for cisplatin resistance. This work nominated 214 genes associated with cisplatin resistance and with high fitscores in ≥2 two chemogenomics studies (including 55 shared between datasets from all three chemogenomics studies) (Figure 1D, dark blue highlighted area). The resulting combined dataset was highly enriched in Gene Ontology (GO) annotations associated with DNA repair, cell cycle, chromosomal organization, and other relevant functions. 54/214 of the identified genes were independently identified in the binary studies as contributing to cisplatin resistance. Merging candidate cisplatin resistance datasets resulted in a list of 286 genes (Figure 1D, Supplementary Table 2).

### Integration based on chemogenomic profile

Proteins that collaborate to execute specific biological processes often show a common pattern of essentiality across multiple growth conditions [36]. As an orthogonal approach to gain insight into cisplatin resistance and DDR functions, we sought to identify genes with overall phenotypic profiles similar to those of well-established, functionally relevant resistance genes. To this end, we analyzed the distribution of the 286 selected candidate genes across co-fitness gene clusters that were based on a comprehensive chemogenomic study, which reported the overall profiles of growth of 4,769 homozygous yeast deletion strains [37] in 418 culture conditions, including treatment with FDA-approved drugs and other bioactives.

**Figure 2:**
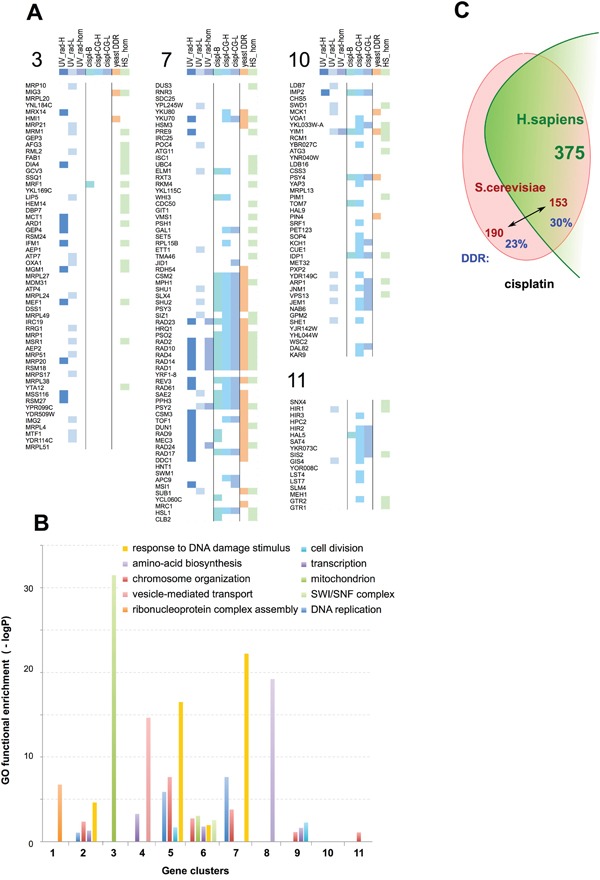
Examples of functional clusters of genes regulating response to UV, X-ray radiation, or cisplatin **A**. Graphical representation of composition and selected properties of clusters (CL) enriched in UV_rad sensitivity-modulating genes (CL 3), in cisplatin sensitivity-modulating genes (CL 10 and 11), or in both (CL 7). Key indicates genes identified as UV_rad HTS≥2 and/or UV_rad LTS (UV_rad-H); or as inducing cisplatin resistance from binary (cispl-B), or chemogenomics (cispl CG-H) with high statistical significance (see text for details). Genes initially defined as lower confidence because of identification from the UV_rad HTS=1 set alone, or only by a single high score for cisplatin sensitization from a single chemogenomics screen, but significantly enriched (p<0.05) within clusters, are denoted as “UV_rad-L” and “cispl-CG-L”, respectively. “Yeast DDR” indicates genes annotated as involved in response to DNA damage; “UV_rad-hom” indicates functional orthologs in fly and/or worm; “HS_hom”, genes have unambiguous orthologs in *H. sapiens*. See Supplementary Table 3 for detailed information on all identified clusters. **B**. Enrichment in Gene Ontology (GO) functions for the identified clusters. **C**. Overall evolutionary conservation of *S. cerevisiae* cisplatin resistance genes in *H. sapiens*. Numbers shown in red font represent individual yeast genes, while green font is used for the number of human genes orthologous to the yeast counterpart. The fraction of yeast genes annotated as involved in DDR is shown in blue.

From this analysis, we identified 11 clusters that were enriched (p≤0.001) for the LTS/HTS/CGS≥2 radiation and/or cisplatin resistance-modulating genes (Figure 2A, 2B, Table 1; extended data in Supplementary Table 3), including two clusters (CL10 and CL11) enriched for cisplatin resistance only. In addition to enrichment for DDR-related gene ontology annotations (CL2, 5-7), some clusters were also enriched in related functions such as chromatin assembly and chromosome organization (CL2, 5-7, 9, 11) (Figure 2B, Table 2). Contrasting the two clusters with the greatest level of enrichment for DDR-related functions (CL5 and CL7), the genes in CL5 provided highly significant resistance not only to multiple platinum compounds, but also to many other classes of DNA damaging agents, including cantharidin, bleomycin, chlorambucil, and others, while those in CL7 were more restricted in activity (Supplementary Table 4).

**Table 1:** Enrichment of candidate gene sets in clusters

Cluster number	UV_rad LTS/HTS≥2	UV_rad HTS=1	cisplatin binary studies	cisplatin chemogenomics studies	Human DDR
1	****	****	*	- /**	
2	***	**		- / ****	
3	****	****			
4	****	****	****	- / ****	
5	****	****	****	**** / ****	****
6	**	****		- /***	
7	****	*	****	**** / ****	****
8	**		**	* / ***	
9	****	***			
10				* / ****	
11				** / ****	

P values indicate the enrichment of each indicated cluster with genes from the indicated data sets, and also likelihood that clusters have human homologues with DDR annotation. *, p<0.05; **, p<0.01; ***, p<0.001; ****, p<0.0001; **** (red font), p<0.00001.

**Table 2:** Enrichment of clusters for Gene Ontology (GO) functions related to DNA damage, cell cycle, and chromosomal organization

Cluster #	2		5		6		7		9		11	
Genes in cluster	28		109		37		67		59		17	
DNA repair	5.0E-06	( 9 )	**4.4E-15**	**( 29 )**			**2.2E-24**	**( 30 )**				
nucleotide-excision repair	1.2E-02	( 3 )					1.3E-07	( 8 )				
double-strand break repair			**8.2E-13**	**( 16 )**			5.3E-07	( 9 )				
meiotic cell cycle			1.3E-06	( 18 )			1.6E-04	( 11 )				
DNA replication	8.6E-02	( 3 )	1.4E-06	( 13 )			2.4E-08	( 12 )				
DNA-dependent ATPase activity			9.8E-10	( 12 )			1.8E-02	( 4 )	9.6E-02	( 3 )		
DNA integrity checkpoint							1.8E-10	( 9 )				
base-excision repair												
DNA recombination			5.8E-10	( 18 )			1.1E-10	( 15 )				
bypass DNA synthesis							6.3E-02	( 2 )				
postreplication repair			6.5E-03	( 4 )								
chromosome organization	4.3E-03	( 7 )	2.4E-08	( 26 )	1.8E-03	( 9 )	1.6E-04	( 14 )	7.4E-02	( 8 )	8.0E-02	( 4 )
structure-specific DNA binding			1.9E-03	( 7 )			7.6E-06	( 8 )				
non-recombinational repair			7.6E-08	( 10 )			1.2E-03	( 5 )				
damaged DNA binding							**2.7E-11**	**( 9 )**				
cell division			2.0E-02	( 7 )					5.6E-03	( 6 )		
response to DNA damage stimulus	2.4E-05	( 9 )	**3.1E-17**	**( 34 )**	1.1E-02	( 7 )	**6.1E-23**	**( 31 )**				
recombinational repair			**5.4E-14**	**( 14 )**			1.3E-07	( 8 )				
SWI/SNF complex					2.8E-03	( 3 )						

P values for the indicated GO function enrichment are shown (with the corresponding number of genes in parentheses). Only clusters with significant (p<0.05) values for the indicated categories are given. Color codes for p-value: light gray, < 1.0E-5; dark grey, < 1.0E-10.

In contrast, for some clusters (e.g., CL3 and 4), the greatest GO terms enrichment reflected processes related to overall cellular robustness (e.g., mitochondrial function or vesicle-mediated transport; Figure 2B); not surprisingly, in chemogenomics profiling these clusters also show overabundance of genes responding to other cellular stresses such as heat sensitivity. Among genes originally nominated from cisplatin resistance sets, fewer were annotated for DDR-related processes compared to genes identified based on resistance to UV/radiation, potentially reflecting the greater diversity of cisplatin resistance mechanisms.

Interestingly, some of the clusters contained more of the genes initially defined as lower confidence, originating from the UV_rad HTS=1 set or only had a single high score for cisplatin sensitization from a single chemogenomics screen, a finding unlikely to have occured by chance (p<0.05; Table 1, Supplementary Tables 1, 3). Although many of the HTS=1 candidates most likely represented false positive hits in functional screening assays, the additional evidence from co-fitness profiling linked some of these genes to the high value set of resistance regulators. Thus, these data suggested an additional 126 yeast genes that may be functionally linked to resistance to DNA damaging agents, albeit with a weaker phenotype (Supplementary Tables 2, 3).

### UV and radiation resistance genes in flies and worms

To provide additional insight, we analyzed resources in FlyBase [38], FlyMine [39], Wormbase [40], and Pubmed to identify genes linked to radiation or cisplatin resistance based on resistance screens performed in the fruitfly *D. melanogaster* (69 genes) or the roundworm *C. elegans* (34 genes) (Supplementary Tables 5–6 and Supplementary Figure 2). Of these, 26/65 genes in *D. melanogaster* and 23/34 in *C. elegans* have orthologs in yeast, of which 14/26 and 15/23, respectively, were also identified in yeast as modulating UV/radiation sensitivity (Supplementary Tables 5–6). The small number of genes thus identified most likely reflects the relatively limited number of screening studies in these organisms specifically focused on DNA damage resistance. Conversely, of the genes shown to increase UV and/or radiation sensitivity in *S. cerevisiae*, 169 were evolutionarily maintained (based on sequence conservation) in *D. melanogaster* and 162 in *C. elegans*, with the majority of the genes (156) conserved in both. However, fewer than 50% of these conserved genes were annotated as relevant to DDR processes (79/169 fly genes and 75/162 worm genes). This lack of annotation mostly likely reflects the much more systematic functional screening efforts in *S. cerevisiae*, although it may also reflect altered activity of these genes in metazoans.

### Conservation of UV, radiation, and cisplatin-resistance genes in humans

**Figure 3:**
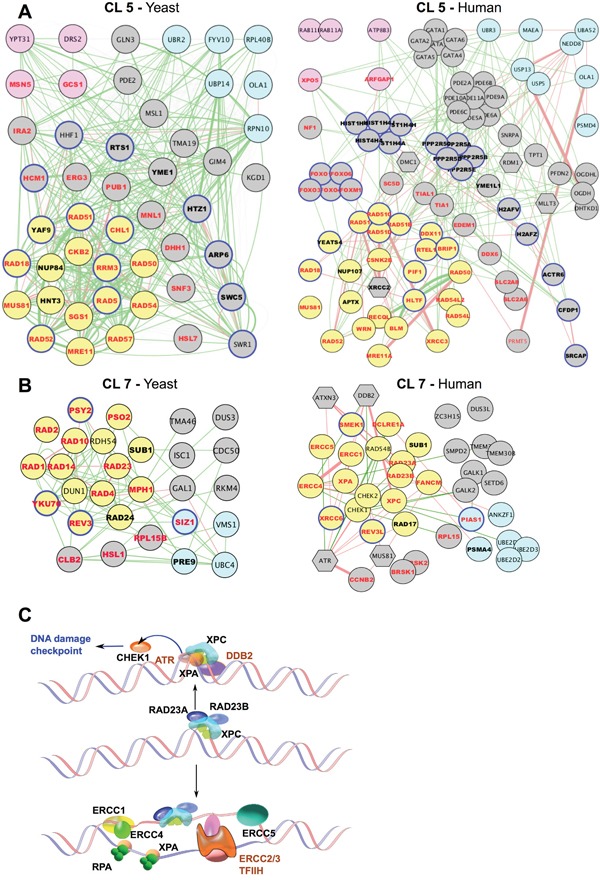
Evolutionary conservation of gene networks related to DDR **A, B**. Gene networks for cluster 5 (A) and cluster 7 (B). For each cluster, networks of both yeast (left) and human (right) orthologous genes are shown. Gene Ontology (GO) functional annotations determined in yeast are indicated (on both yeast and human orthologs): DDR, yellow node fill; transport/secretion, pink node fill; proteosome/ubiquitination/degradation, light-blue node fill; chromosome/chromatin association, blue node outline. Bold font, cisplatin- and UV_rad sensitizing candidates; red font: cisplatin-sensitizing. Edges: physical interaction (red) or signaling interaction (green). Functionally connected, but not orthologous to yeast counterparts in the corresponding clusters, human genes are shown as hexagons. **C**. Schematic representation of defined components of the Nucleotide Excision Repair NER) pathway enriched in cluster 7 (black font) and associated signaling partners (brown).

We next identified the human orthologs of genes annotated as relevant to UV, radiation, or cisplatin resistance in yeast (Figure 2C). Because of gene duplication and other events, in some cases individual genes in yeast are represented by paralogous gene families in humans, gene-ortholog assignment was inexact; however, approximately 45% of yeast genes linkage to UV, radiation, or cisplatin resistance with high confidence, had definable human orthologs or paralog sets. The fraction of *S. cerevisiae* genes identified through primary resistance to UV/radiation or cisplatin had similar frequencies for human orthologs. Side-by-side juxtaposition of genes in yeast CL5 (Figure 3A, left) and CL7 (Figure 3B, left) with human orthologs (Figure 3A, 3B, right) emphasizes that most of the genes thus identified in yeast are conserved in humans. Among the human orthologs, 20% are functionally annotated in Ensemble (release 84, 2016) as having DDR-related functions, particularly for orthologs of yeast genes identified for roles in regulating UV or radiation responses (Supplementary Figure 2). These included highly validated proteins with homologs such as ERCC1, ERCC4, XRCC3, and RAD54. Figure 3C illustrates a subset of genes in the nucleotide excision repair (NER) complex, enriched in CL7. Overall, this analysis based on yeast, fly and worm genes implicated 684 human genes as potentially involved in resistance to UV, radiation, or cisplatin (Supplementary Table 7).

### RNAi assessment of roles for candidate genes involved in cisplatin resistance in human cancer models

**Figure 4:**
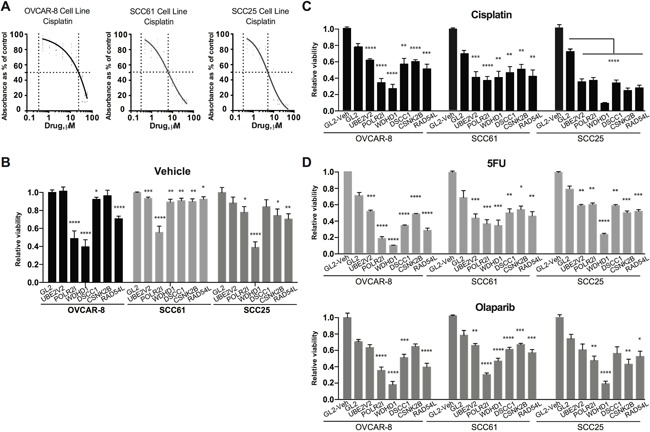
Evaluating candidate gene regulation of sensitivity to cisplatin treatment **A**. IC50 determination for cisplatin for the OVCAR-8, SCC61, and SCC25 cell lines. **B**. Relative viability of OVCAR-8, SCC61, and SCC25 cell lines assessed by CellTiterBlue (CTB) following treatment with negative control siRNA (GL2) or siRNAs targeting the indicated genes. Data are normalized to CTB values for GL2. **C**. Relative viability of cells treated with the indicated siRNAs 72 hours after treatment with IC20-30 levels of cisplatin. GL2/vehicle treated cells are included as reference. **D**. Data as for C., except following treatment of cells with 5-FU (top) or olaparib (bottom). *, P < 0.05, **, P <0.01, ***, P <0.001, ****, P <0.0001 for all graphs.

We sampled genes identified from conservation with functionally defined yeast genes, choosing 5 from clusters and one not (UBE2V2), for direct evaluation for roles in cisplatin resistance in human cells. Head and neck cancers (HNCs), and epithelial ovarian cancers (EOCs) are commonly treated with cisplatin and other platinum-based compounds [8, 41]. We therefore used the cisplatin-resistant serous EOC cell line OVCAR-8, and two HNC cell lines, SCC61 and SCC25, as models. For each cell line, we used two pooled small interfering RNAs (siRNAs) to deplete a positive control gene (REV3L, previously defined as contributing to cisplatin resistance [10]), a negative control scrambled siRNA (siGL2), or genes identified in our analysis (Figure 4, Supplementary Table 8) but never previously defined as regulating cisplatin sensitivity in humans. 24 hours after transfection, cells were treated with vehicle, or an IC20-IC30 concentration of cisplatin previously established for each cell line (Figure 4A). As all of the genes were evolutionarily conserved and often associated with biologically essential functions, we first established the intrinsic effect of each siRNA pool on fundamental cell viability using CellTiterBlue (Figure 4B). At 72 hours after vehicle treatment, three genes (POLR2I, RAD54L, and WDHD1) significantly reduced viability (by 30-60%) in 2 of the 3 cell lines tested, while 3 genes (UBE2V2, DSCC1, and CSNK2B) had little or no effect on viability.

We then gauged the ability of each siRNA pool to sensitize cells to cisplatin (Figure 4C), 72 hours after drug treatment. Only a limited sensitization effect was observed in the cisplatin-resistant OVCAR-8 line with depletion of UBE2V2, DSCC1, and CSNK2B. In contrast, depletion of UBE2V2, DSCC1, and CSNK2B had a marked sensitization effect in SCC61 and SCC25 cells. In addition, depletion of POLR2I, RAD54L, and WDHD1 each had a sensitizing effect in these cell lines, independent of their fundamental role in viability. As a control for specificity, we used qRT-PCR, confirming that individual siRNAs efficiently depleted their target mRNAs (Supplementary Figure 3). We also asked if the sensitization phenotypes observed were specific to treatment of cells with cisplatin, or whether depletion of the selected genes sensitized cells to additional drugs that caused DNA damage. 5-fluorouracil (5-FU) causes DNA damage by inducing unbalanced replication due to depletion of thymine pools, and olaparib causes DNA damage by inhibiting poly-ADP ribose polymerase (PARP1) [42, 43]. We found the pattern of sensitization to these drugs closely paralleled that seen with cisplatin; notably, depletion of WDHD1 had a very striking effect on sensitization to the PARP1 inhibitor olaparib (Figure 4D).

### Cisplatin sensitivity candidate genes influence magnitude and duration of *γ* H2AX foci formation induced by cisplatin

**Figure 5:**
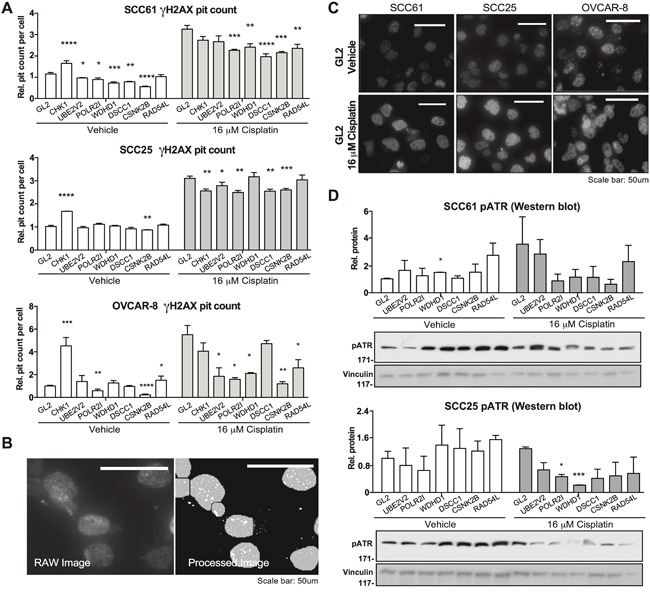
Evaluation of candidate gene regulator of DNA damage responses **A**. Quantification of number of *γ*H2AX-positive foci per cell nucleus for cells transfected with negative control GL2 siRNA, or siRNA targeting indicated genes, 18 hours following treatment with vehicle (DMSO) or cisplatin as indicated. Data are normalized to values for GL2-transfected cells. **B**. *γ*H2AX foci were quantified by automated scoring using MetaXpress software. Image shows representative raw and processed images used for quantitation. **C**. Representative images for vehicle-versus cisplatin-treated cells for the indicated cell lines, as quantified in A. **D**. Representative images and quantification for phosphorylated ATR. *, P < 0.05, **, P <0.01, ***, P <0.001, ****, P <0.0001 for all graphs.

Cisplatin triggers a DNA damage response characterized by the formation of phosphorylated histone H2AX (*γ* H2AX)-positive foci at the site of DNA damage [44, 45], dependent on the action of ATR and associated with the induction of downstream CHEK1 or CHEK2 kinases [46]. We investigated whether the candidate gene set impaired appearance of *γ* H2AX foci following cisplatin treatment (Figure 5A-5C). After depleting candidate or control genes for 24 hours, we treated SCC61, SCC25, or OVCAR-8 cells for 18 hours with vehicle or 16 μM of cisplatin, and then assessed the degree of *γ* H2AX foci formation. A CHEK1-depletion control showed elevated foci formation in vehicle-treated cells, reflecting induction of a DNA damage response, in agreement with previous reports [47]. In contrast, depletion of the sensitization candidate genes either indicated no effect, or reduction in basal levels of DNA damage foci in vehicle-treated cells (Figure 5).

We then examined *γ* H2AX foci formation induced by cisplatin. In this context, the CHEK1-control depleted cells had fewer foci than GL2/vehicle-treated cells, reflecting the uncoupling of the DNA damage response signaling system. Strikingly, all 6 of the genes of interest significantly reduced cisplatin-induction of *γ* H2AX foci in at least 2 of 3 cell models. Some of the most striking effects were observed in the cisplatin-resistant OVCAR-8 cell model, with depletion of UBE2V2 and WDHD1 almost eliminating cisplatin response, in spite of having no effect on basal levels. These results suggested potential defects in DNA damage response signaling associated with depletion of the genes of interest. Supporting this interpretation, Western analysis of control or gene-depleted cells treated with vehicle or cisplatin for 18 hours showed distinct patterns of phosphorylated (active) ATR (Figure 5D). Referenced to vehicle-treated, GL2-depleted cells, depletion of WDHD1, RAD54L, and CSNK2B elevated basal activation of ATR; in contrast, in cells treated with cisplatin, the induction of phospho-ATR was significantly reduced following depletion of WDHD1, DSCC1, CSNK2B, POLR2I, and RAD54L.

**Figure 6:**
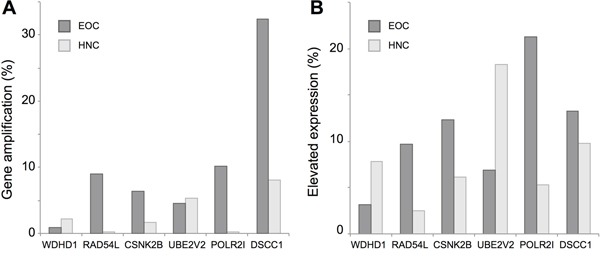
Copy number variation (CNV) and genes expression for cisplatin resistance genes based on TCGA profiling **A**. Percent of tumors with elevated copy number of the indicated genes, based on analysis of 488 HPV-HNCs (light gray) and 540 EOCs (dark gray) in the TCGA data set. **B**. Percent HPV- HNC or EOC tumors with elevated expression (z score >2) in the TCGA dataset.

Finally, the Cancer Genome Atlas (TCGA) reports genomic and transcriptomic data on 530 HNCs (including 488 human papillomavirus negative (HPV-) HNCs) [48–50] and 540 EOCs [51], with additional information available via cBioPortal [52]. Analyzing this data, we found gene amplification (Figure 6A) and overexpression (Figure 6B) of WDHD1, RAD54L, CSNK2B, UBE2V2, POLR2I and DSCC1 in a significant subset of these tumors, suggesting variation in the expression of these genes might contribute to innate resistance to cisplatin, radiation therapy, and other DNA damaging agents.

## DISCUSSION

In summary, this study systematically mined large scale datasets to generate a comprehensive set of *S. cerevisiae* genes that functionally influence resistance to cisplatin, UV, and radiation; we then used clustering and analysis of evolutionary conservation to map this set of genes to human orthologs. The ultimate goal of this work was to evaluate whether functional analysis of genes in yeast and other lower eukaryotes could help identify human genes important for the resistance to DNA-damaging drugs commonly used to treat cancer, particularly cisplatin. The generated datasets (Supplementary Tables 1–2, 7) represent a novel and useful resource for the field.

By integrating multiple datasets linked to DDR responses, we identified a group of genes that affect these processes and have been highly validated in lower eukaryotes, with a subset of these genes segregating into clusters with specific chemogenomic profiles. Human orthologs of genes in these clusters include many already highly validated for roles in DNA repair, such as ERCC1/4/5, CHEK1/2, WRN, BLM, and others. The identified clusters also include genes annotated for DNA repair in yeast but not in humans, and genes such as PIAS1, human ortholog of SIZ1, which may influence activity of DNA repair proteins through a SUMOylation mechanism [53]; BRSK1, ortholog of HSL1, a little studied kinase typically considered a regulator of spindle-formation and centrosomes, although noted in one older study as potentially involved in an alternative UV checkpoint [54]; and the genes POLR2I, WDHD1, CSNK2B and DSCC1 (orthologs of RPB9, CTF4, CKB2 and DCC1), investigated functionally in human cells here. Interestingly, these clusters also contained a number of genes with similar chemogenomic phenotypes that were not annotated for roles in DDR in humans or yeast: these gene pairs, such as ISC1/SMPD2, RKM4/SETD6, and others, are candidates for future evaluation.

Subsequent direct evaluation of a set of human orthologs of genes selected to contain some from within clusters, and some not part of clusters, indicated that a number of them influenced resistance to multiple DNA damaging agents. Of the specific genes we sampled, some have known roles as components of specific DNA repair machineries. For example, UBE2V2 is a variant ubiquitin-conjugating E2 enzyme that is orthologous to the yeast MMS2 gene, implicated in HR and translesion synthesis, that has been shown to promote UV resistance in human cells, with overexpression linked to poor prognosis in some cancer types [55]. RAD54L is a DEAD-like helicase/translocase that has been shown to function as part of the overall machinery for HR-based repair and genome stabilization [56]. In contrast, DSCC1 (also known as hDCC1) is known to be a component of the replication factor C (RFC) complex that contributes to DNA replication, and to have phenotypes related to DNA cohesion [57, 58]; however, it has never been implicated in cisplatin resistance. Similarly, WDHD1 is a WD40-domain rich protein that has been implicated in interaction with and regulation of the pre-replicative complex in human cells, induced during genotoxic stress, making it a plausible regulator of sensitization to DNA damage [59, 60]. WDHD1 has also never been shown to influence cisplatin resistance.

Together, this work emphasizes the growing realization that DDR-relevant functions can be disseminated across broad networks of genes that are not obviously related to the core DDR machinery. As genomic testing becomes standard in the clinic, a major challenge has been to use this information to develop prognostic markers, and identify actionable therapeutic targets, to improve clinical outcomes. Recent studies have catalogued mutations in defined DDR genes in cancer, correlating mutations or expression changes in these genes with genomic instability and gene expression changes that predict response to current therapies [61]. Collectively, this work has led to clinical gains, identifying proteins that can be therapeutically targeted alone or in synthetically lethal combinations with other drugs, or specific DNA lesions. The exceptional responses of patients with BRCA mutations to olaparib and other PARP1-targeting drugs provide a clear example of the utility of such an approach [62]. Such work also has potential importance in identifying patients who might respond to newer treatment options such as immunotherapies, given the appreciation of the importance of tumor-specific mutated proteins that arise from a high mutational burden in providing epitopes that can be recognized by the immune system [63, 64].

While much work has focused on study of genes most directly related to core machineries related to HR, MMR, NHEJ, and other specific DNA repair processes, a number of recent studies have illustrated functionally important control of activity of repair proteins by signaling systems previously thought to function independently. Additional control of the repair process may be mediated in part by epigenetic regulators or microRNAs (miRs) [65, 66]. For example, the recent recognition that androgen signaling regulates expression of a suite of DDR genes accentuates the importance of considering non-canonical candidates for control of therapy resistance [67]. The model provided by this study suggests an orthogonal approach to understand this type of biological network. However, these data also emphasize the challenges of developing reliable prognostic biomarkers, or identifying unique, targetable DDR genes to improve cisplatin response, when large numbers of genes each make incremental contributions to treatment resistance phenotypes. It is likely that, as more chemogenomics, protein-protein and genetic studies become available (see [68]), a similar integrative approach will identify additional genes beyond the networks described here. Additionally, assessment of epigenetic changes, including DNA methylation [69] and gene silencing [70] for cisplatin resistance in the context of distinct tumor subtypes may yield different patterns of dependence, based on which genes are expressed in which tumor subclasses. For example, even among HNCs, the HPV-negative subtype analyzed here has features that distinguish it from HPV-positive disease [71, 72]. Further work is clearly needed.

## MATERIALS AND METHODS

*Detailed methods for analysis of datasets, performance of siRNA analysis, and antibodies for Western blotting are found in Supplementary Materials Online Methods*.

### Identification of genes and human orthologs relevant to radiation and/or cisplatin resistance in model organisms

*S. cerevisiae* genes with loss-of-function phenotypes of reduced resistance to radiation or cisplatin were identified from the Saccharomyces Genome Database (SGD, accessed 02/02/2015) [21], and integrated with data manually curated from published screens. Genes relevant to radiation and/or cisplatin resistance in *D. melanogaster* and *C. elegans* were extracted from FlyBase [38], and WormBase [40], respectively. Human orthologs of genes defined through analysis of *S. cerevisiae*, *D. melanogaster*, and *C. elegans* were obtained through batch searches using Ensemble Biomart (http://useast.ensembl.org/biomart/martview/) [73].

### Clustering, functional enrichment, and TCGA analysis

Data corresponding to the chemogenomic analysis of a homozygous deletion mutant collection were retrieved from the supplementary materials of Hillenmeyer et al [37] and imported in Multiple experiment Viewer (MeV, [74]). Following the optimal gene function-predicting strategy identified in ref [37], we have employed unsupervised complete-linkage hierarchical cluster analysis [74], using uncentered Pearson correlation. Clusters containing more than 15 genes (arbitrary size selection), and containing three or more UV_rad or cisplatin resistance genes were identified, and the statistical significance of enrichment of these clusters for the UV_rad and/or cisplatin sensitivity mutants was calculated using a hypergeometric distribution test. Functional enrichment of clusters was analyzed using DAVID [75, 76], normalized to results from the complete list of 4,769 genes for which homozygous *S. cerevisiae* single deletion strains are available. A threshold of 15 genes for minimal cluster size was selected to allow statistical significance in estimations of gene function enrichment. For TCGA analysis, the most recent datasets for ovarian serous cystadenocarcinoma and head and neck squamous cell carcinoma (as of August 25, 2016) were accessed and analyzed using tools available at cBioPortal for Cancer Genomics (http://www.cbioportal.org/, [52]).

### Cell culture

SCC25 SCCHN cells were obtained from the American Type Culture Collection (ATCC). SCC61 SCCHN cells and the ovarian carcinoma cell line OVCAR8 were obtained from the FCCC Cell Culture Facility. Authentication of all cell lines by genotyping was performed by IDEXX BioResearch (Columbia, MO). SCC61 and SCC25 cell lines were cultured in DMEM-F12 media containing 10% fetal bovine serum (FBS), L-glutamine (L-glu) and penicillin/streptomycin (pen/strep). OVCAR8 was cultured in RPMI-1640 media containing 10% FBS, L-glu and pen/strep.

### siRNA drug sensitization and validation

Human genes to be assessed for modulation of cisplatin sensitivity were depleted using siRNAs from Qiagen (Hilden, Germany) with positive and negative controls for transfection and normalization. After 24 hours recovery, cells were treated with cisplatin or vehicle for 72 hours, cell viability measured using a Cell Titer Blue assay (Promega, Madison, WI ), and sensitization index (SI) determined. For each gene of interest, siRNA sensitization assays were initially performed with 4 independent siRNAs; subsequently, the two best performing RNAs (Supplementary Table 8) were pooled and used for functional testing. For evaluation of depletion efficacy, at 48 hours after transfection, total RNA was extracted using the RNeasy Mini Kit (Qiagen, Hilden, Germany), reverse-transcribed using standard approaches and analyzed by Taqman chemistry using Assay-on-Demand (Supplementary Table 9). To explore specificity of genes for response to cisplatin, 24 hours post transfection with pooled siRNAs, cells were treated in parallel with selected concentrations of cisplatin, 5-fluorouracil (5-FU), paclitaxel, olaparib or vehicle. All drugs, except olaparib, were obtained in Fox Chase Cancer Center Pharmacy. Olaparib was purchased from LC Laboratories, Woburn, MA. After 72 hours, a CellTiterBlue assay was performed following the manufacturer's protocol, and the SI was determined for each drug as previously described [18].

### Automated immunofluorescence detection of *γ*-H2AX

The quantitative assay of formation of foci containing phosphorylated histone H2AX (*γ*-H2AX) in SCC25, SCC61 and OVCAR8 cells was performed in cells transfected in triplicate in 96 well plates. CHEK1-targeting siRNA was used as a positive control (M-003255-04-0005 GE-Dharmacon, Lafayette, CO), and siGL2 as a normalization control. 24 hours post transfection cells were treated with 16 or 30 μM cisplatin or with vehicle. After 18 hours, cells were washed with ice-cold PBS, fixed with 4% PFA for 10 min, washed again, permeabilized with 0.1% Triton-X100 and stained with anti-*γ*H2AX primary antibodies (1:1000, Mouse Monoclonal, Millipore Upstate, Billerica, MA) overnight at +4C°, followed by staining with FITC-tagged secondary antibodies (1:1000, goat anti-mouse IgG (H+L), Alexa Fluor^®^ 488 conjugate) for 1 hour at room temperature. 9 independent image fields from each individual well were acquired with an automated high-throughput screening–microscope (ImageXpress micro, Molecular Device Sunnyvale, CA), driven by MetaXpress software (Molecular Devices, Sunnyvale, CA). Images acquired from immunofluorescent samples were analyzed utilizing the Transfluor analysis module of MetaXpress allowing for quantitation of stained foci within nuclear segmentation. Automated cell counts and recognition were based on DAPI stained nuclear segmentation, with the *γ*-H2AX staining score based on the quantitation of foci using the ‘pit count per cell’ parameter. Results from these analyses were displayed within Acuity (Molecular Devices, Sunnyvale CA) and Microsoft Excel.

### Western blot analysis

SCC61 and SCC25 cells were transfected in 6 well plates, treated with 16uM Cisplatin for 18 hours and lysed in CelLytic MT Cell Lysis Reagent (Sigma-Aldrich, St. Louis, MO). Protein concentrations of the resulting lysates were measured using the Pierce BCA Protein Assay Kit (Thermo Scientific, Waltham, MA). Western Blotting was performed using standard procedures, and blots developed by chemiluminescence using Luminata Western HRP substrates (Classico, Crescendo and Forte, EMD Millipore). anti-phospho-ATR (Ser428) rabbit, polyclonal primary antibody (#2853) was provided by, Cell Signaling, Danvers, MA. Quantification of signals on Western blots was done using the NIH ImageJ Imaging and Processing Analysis Software with signaling intensity normalized to loading control.

## SUPPLEMENTARY MATERIALS FIGURES AND TABLES
















