# Supplementary material for: Identification of evolutionarily conserved DNA damage response genes that alter sensitivity to cisplatin
Source: Oncotarget. 2016 Nov 15;8(12):19156–71. doi: 10.18632/oncotarget.13353 (PMC5386675; doi:10.18632/oncotarget.13353)
Supplement: Supplementary file 1 [file oncotarget-08-19156-s001.pdf]

Identification of evolutionarily conserved DNA damage response genes that alter sensitivity to cisplatin

SUPPLEMENTARY DATA

SUPPLEMENTARY MATERIALS AND METHODS

Identification of genes regulating radiation and/or cisplatin resistance in yeast and other lower eukaryotes

The Saccharomyces Genome Database (SGD, [1]; data current on Dec-28-2015) was searched for a list of phenotypes including "gamma ray resistance", "X ray resistance", "ionizing radiation resistance," and "UV resistance". Identifiers for genes, mutations in which resulted in altered phenotypes based on these search terms, were extracted and pooled. In parallel, the SGD was searched for the phenotype of altered "resistance to chemicals (cisplatin)", and identifiers for genes in which inactivating mutation or deletion resulted in increased sensitivity to cisplatin were extracted. An initial assessment of high-throughput studies (defined here as those screening > 15% of the genome) reported in SGD revealed inconsistencies in the ways their results were transferred to SGD. Therefore, we also reviewed a number of primary papers reporting high throughput studies from Pubmed [2-13]. Data available in these papers were extracted manually and integrated with the results reported in SGD.

To sort chemogenomics (CGS) data, the sensitization gradient observed for cisplatin in each of 3 independent screens was aligned with the set of high confidence hits determined through performance of binary screens (HTS and/or LTS reported at least two independent studies). The set of CGS genes ranging from most sensitizing to less sensitizing, with a cut-off at the point where >65% of high confidence binary genes had been observed in CGS analysis, was taken for further consideration as a high confidence CGS set for each independent screen. Subsequently, intersections between each pair of high-confidence subsets from the 3 screens were established: genes found in at least 2 screens were considered reproducible. A hypergeometric test was used to establish the statistical significance of each intersection ($p<1$ E-10). The three candidate lists were then merged into one high-confidence CGS candidate set.

In additional analysis, FlyBase (http://flybase.org, [14]) and FlyMine (http://www.flymine.org/, [15]) were searched for the phenotype "radiation sensitive" and for the GO Term GO:0009411, "response to UV". WormBase (http://www.wormbase.org, [16]) was searched through WormMine (http://im-dev.wormbase.org/tools/wormmine/) using queries for mutations and RNAi-downregulated genes resulting in the phenotypes "gamma radiation hypersensitive"; "ionizing radiation hypersensitive"; "X-ray radiation hypersensitive"; "UV radiation". Data were merged with gene list with a review on DNA damage response in C. elegans [17].

For characterization of yeast clusters of interest, a t-test was used to identify which screening modalities (i.e., response to which individual drugs) differed significantly between the selected cluster of interest and the rest of the yeast clones, based on level of sensitivity, using data in Hillenmeyer et al. [18]. For each statistically significant category (drug), the number of experiments which show the difference in sensitivity was extracted separately and sorted into two bins: Highly significant (t-test < 1.010E-7) and moderately significant (t-test between 1.010E-2 and 1.010E-7). Mechanism of action was extracted for each drug using online resources: The NCI Drug Dictionary (http://www.cancer.gov/publications/dictionaries/cancer-drug) and The DrugBank (http://www.drugbank.ca).

siRNA screening

Human genes to be assessed for modulation of cisplatin sensitivity were depleted using two pooled siRNAs from Qiagen (Hilden, Germany) per gene. siRNAs targeting polo-like kinase 1 (*PLK1*) were used as a positive control for transfection, and scrambled siRNAs targeting the firefly luciferase gene (GL2) were included as a negative control for normalization (Dharmacon, Pittsburgh, PA). SiRNAs were introduced into cells by reverse transfection, using DharmaFECT-1 (Dharmacon, Pittsburgh, PA) diluted in reduced-serum media (OptiMEM, Invitrogen) in V-bottom 96-well dilution plates containing siRNA pools using a bulk reagent microplate dispenser. After 30 min at room temperature, each siRNA-lipid complex was aliquoted into 96-well flat-bottom test plates using a CyBio Vario liquid handler, followed by addition of cells in normal growth media lacking antibiotics (10,000 cells/well for SCC61 and SCC25, 4,000 cells/well for OCAR8, 90 µl final volume/well).

After 24 hours recovery, cells were treated with cisplatin or vehicle for 72 hours, then cell viability measured using a Cell Titer Blue assay (Promega, Madison, WI), with signal quantified after 3 hours using a Envision (Perkin Elmer, Waltham, MD, USA) multi-label microplate reader. To calculate cell viability

following siRNA treatment, the fluorescence intensity (FI) value from each well targeted by gene-specific siRNAs was divided by the mean FI value from three reference wells containing the non-targeting negative control GL2 siRNA on each plate to yield a viability score (V) defined as V = (fluorescence intensity, query gene-specific siRNA)/(mean fluorescence intensity, GL2 siRNA)) corresponding to each gene. The sensitization index (SI) of each siRNA was then defined as the viability of cells in the presence of siRNA and drug divided by the viability of the cells in the presence of siRNA and vehicle (SI = (VsiRNA + drug)/(VsiRNA + vehicle)). Biological significance was defined as a decrease or increase in the SI greater than 15%, as in previous studies [19]. These experiments were performed at least 3 times independently for each cell line. Four siRNAs were tested for each gene; for validation of on-target activity, the two siRNAs with the most robust sensitizing phenotype were tested for depletion of each gene by RT-PCR, and then were pooled together for further experiments (Supp Tables S8, S9).

Antibodies for western analysis

Primary antibodies were used in 1:1000 dilution (if not indicated differently) and included: anti-phospho-ATR (Ser428) (rabbit, polyclonal, #2853, Cell Signaling, Danvers, MA), anti-ATR (rabbit, polyclonal, ab10312, Abcam, Cambridge, MA), anti-phospho-p53 (Ser15) (rabbit, polyclonal, #9284, Cell Signaling), anti-p21 Waf/Cip1 (rabbit, monoclonal, #2947, Cell Signaling, Danvers, MA), anti-vinculin (mouse, monoclonal hVIN-1, #V9131, Sigma-Aldrich, St. Louis, MO). Secondary anti-mouse and anti-rabbit HRP-conjugated antibodies (GE Healthcare, Little Chalfont, UK) were used at a dilution of 1:10,000.

Interaction networks of yeast genes and their human orthologs

Human orthologues for yeast genes of interest were identified using Ensemble Biomart (http://useast.ensembl.org/biomart/martview/) [20], using the orthology confidence cutoff 1 (maximum stringency). Gene homology was further verified using the P-POD: Princeton Protein Orthology Database (http://ppod.princeton.edu) [21]. For both yeast and human proteins, interaction networks were built using Cytoscape [22] with a GeneMania plugin [23], with the types of interactions restricted to physical and genetic. For human genes, settings allowed retrieval of up to five additional genes, to provide biological context.

Comprehensive genomic profiles are available for SCC25 and OVCAR-8 cell lines, including mutational landscape and gene amplification/deletion profiles, at the following links:

http://www.cbioportal.org/case.do?cancer_study_id=cellline_ccle_broad&case_id=OVCAR-8 and http://www.cbioportal.org/case.do?cancer_study_id=cellline_ccle_broad&case_id=SCC-25

Only limited details are available in regard of SCC61, as described at [24] and http://cancer.sanger.ac.uk/cosmic/sample/overview?id=1122673. In general, the comparison between the genomic profiles of HNSCC cell lines with the genomic profiles of HNSCC tumors indicates a good correlation [25].

Rank calculation for human orthologs of genes modulating UV_rad and/or cisplatin resistance in model organisms (Table S7)

The rank for each human candidate gene was calculated based on two criteria: 1) the confidence in identification of a candidate gene in a model organism, and 2) the confidence in identification of the corresponding human ortholog (s). Confidence in identification of a UV_rad candidate gene in a model organism was calculated based on the number of independent publications implicating the gene in both LTS and HTS datasets, as follows: $0.5*(n(LTS)_i/N(LTS) + n(HTS)_i/N(LTS))$ where n_i is the number of publications supporting the candidate, and N is the maximum number publications supporting a candidate in the gene set. Where the assignment of the gene was also supported by data from at least one other model organism, the confidence was set to 1. For the cisplatin set, the confidence was calculated in the same way, except that instead of using LTS and HTS counts, "binary" and "CGS" counts were used (i.e., $0.5*(n(binary)_i/N(binary) + n(CGS)_i/N(CGS))$. Thus, genes implicated by only one HTS or CGS publication coupled with functional clustering with better-characterized genes have the lowest confidence.

Calculation of the confidence in identification of appropriate human ortholog(s) for yeast genes incorporated consideration of the overall degree of evolutionary conservation (e.g., existence of orthologs in D.melanogaster and C. elegans), and the percent identity between the genes in model organisms and humans. The formula (0.1* C(hs) +0.1* C(dm) 0.1* C(ce) + 0.65*P(hom)) was used, where C(hs), C(dm), C(ce) are confidence scores for H. sapiens, D. melanogaster and C. elegans orthologs (either 1 or 0), as retrieved using Ensemble Biomart [20]. P(hom) is the percent of sequence identity between the model organism and human proteins. The final rank was assigned by combining the confidence and orthology scores, and subsequently calculating rank for each value in the set.

REFERENCES

1. Cherry JM, Hong EL, Amundsen C, Balakrishnan R, Binkley G, Chan ET, Christie KR, Costanzo MC, Dwight SS, Engel SR, Fisk DG, Hirschman JE, Hitz BC, et al. Saccharomyces Genome Database: the genomics resource of budding yeast. Nucleic Acids Res. 2012; 40: D700-5.
2. Begley TJ, Rosenbach AS, Ideker T, Samson LD. Damage recovery pathways in Saccharomyces cerevisiae revealed by genomic phenotyping and interactome mapping. Molecular cancer research : MCR. 2002; 1: 103-12.
3. Begley TJ, Rosenbach AS, Ideker T, Samson LD. Hot spots for modulating toxicity identified by genomic phenotyping and localization mapping. Molecular cell. 2004; 16: 117-25.
4. Bennett CB, Lewis LK, Karthikeyan G, Lobachev KS, Jin YH, Sterling JF, Snipe JR, Resnick MA. Genes required for ionizing radiation resistance in yeast. Nature genetics. 2001; 29: 426-34.
5. Birrell GW, Giaever G, Chu AM, Davis RW, Brown JM. A genome-wide screen in Saccharomyces cerevisiae for genes affecting UV radiation sensitivity. Proc Natl Acad Sci U S A. 2001; 98: 12608-13.
6. Game JC, Birrell GW, Brown JA, Shibata T, Baccari C, Chu AM, Williamson MS, Brown JM. Use of a genome-wide approach to identify new genes that control resistance of Saccharomyces cerevisiae to ionizing radiation. Radiation research. 2003; 160: 14-24.
7. Giaever G, Flaherty P, Kumm J, Proctor M, Nislow C, Jaramillo DF, Chu AM, Jordan MI, Arkin AP, Davis RW. Chemogenomic profiling: identifying the functional interactions of small molecules in yeast. Proceedings of the National Academy of Sciences of the United States of America. 2004; 101: 793-8.
8. Hanway D, Chin JK, Xia G, Oshiro G, Winzeler EA, Romesberg FE. Previously uncharacterized genes in the UV- and MMS-induced DNA damage response in yeast. Proceedings of the National Academy of Sciences of the United States of America. 2002; 99: 10605-10.
9. Liao C, Hu B, Arno MJ, Panaretou B. Genomic screening *in vivo* reveals the role played by vacuolar H+ ATPase and cytosolic acidification in sensitivity to DNA-damaging agents such as cisplatin. Molecular pharmacology. 2007; 71: 416-25.
10. Wu HI, Brown JA, Dorie MJ, Lazzeroni L, Brown JM. Genome-wide identification of genes conferring resistance to the anticancer agents cisplatin, oxaliplatin, and mitomycin C. Cancer research. 2004; 64: 3940-8.
11. Xia L, Jaafar L, Cashikar A, Flores-Rozas H. Identification of genes required for protection from doxorubicin by a genome-wide screen in Saccharomyces cerevisiae. Cancer Res. 2007; 67: 11411-8.
12. Birrell GW, Brown JA, Wu HI, Giaever G, Chu AM, Davis RW, Brown JM. Transcriptional response of Saccharomyces cerevisiae to DNA-damaging agents does not identify the genes that protect against these agents. Proc Natl Acad Sci U S A. 2002; 99: 8778-83.
13. Lee W, St Onge RP, Proctor M, Flaherty P, Jordan MI, Arkin AP, Davis RW, Nislow C, Giaever G. Genome-wide requirements for resistance to functionally distinct DNA-damaging agents. PLoS Genet. 2005; 1: e24.
14. Attrill H, Falls K, Goodman JL, Millburn GH, Antonazzo G, Rey AJ, Marygold SJ, FlyBase c. FlyBase: establishing a Gene Group resource for Drosophila melanogaster. Nucleic Acids Res. 2016; 44: D786-92.
15. Lyne R, Smith R, Rutherford K, Wakeling M, Varley A, Guillier F, Janssens H, Ji W, McLaren P, North P, Rana D, Riley T, Sullivan J, et al. FlyMine: an integrated database for Drosophila and Anopheles genomics. Genome Biol. 2007; 8: R129.
16. Howe KL, Bolt BJ, Cain S, Chan J, Chen WJ, Davis P, Done J, Down T, Gao S, Grove C, Harris TW, Kishore R, Lee R, et al. WormBase 2016: expanding to enable helminth genomic research. Nucleic Acids Res. 2016; 44: D774-80.
17. Lans H, Vermeulen W. Nucleotide Excision Repair in Caenorhabditis elegans. Mol Biol Int. 2011; 2011: 542795.
18. Hillenmeyer ME, Ericson E, Davis RW, Nislow C, Koller D, Giaever G. Systematic analysis of genome-wide fitness data in yeast reveals novel gene function and drug action. Genome biology. 2010; 11: R30.
19. Astsaturov I, Ratushny V, Sukhanova A, Einarson MB, Bagnyukova T, Zhou Y, Devarajan K, Silverman JS, Tikhmyanova N, Skobeleva N, Pecherskaya A, Nasto RE, Sharma C, et al. Synthetic lethal screen of an EGFR-centered network to improve targeted therapies. Sci Signal. 2010; 3: ra67.
20. Yates A, Akanni W, Amode MR, Barrell D, Billis K, Carvalho-Silva D, Cummins C, Clapham P, Fitzgerald S, Gil L, Giron CG, Gordon L, Hourlier T, et al. Ensembl 2016. Nucleic Acids Res. 2016; 44: D710-6.
21. Heinicke S, Livstone MS, Lu C, Oughtred R, Kang F, Angiuoli SV, White O, Botstein D, Dolinski K. The Princeton Protein Orthology Database (P-POD): a comparative genomics analysis tool for biologists. PLoS One. 2007; 2: e766.
22. Shannon P, Markiel A, Ozier O, Baliga NS, Wang JT, Ramage D, Amin N, Schwikowski B, Ideker T. Cytoscape: a software environment for integrated models of biomolecular interaction networks. Genome Res. 2003; 13: 2498-504.
23. Warde-Farley D, Donaldson SL, Comes O, Zuberi K, Badrawi R, Chao P, Franz M, Grouios C, Kazi F, Lopes

CT, Maitland A, Mostafavi S, Montojo J, et al. The GeneMANIA prediction server: biological network integration for gene prioritization and predicting gene function. Nucleic Acids Res. 2010; 38: W214-20.

24. Young NR, Liu J, Pierce C, Wei TF, Grushko T, Olopade OI, Liu W, Shen C, Seiwert TY, Cohen EE. Molecular phenotype predicts sensitivity of squamous cell carcinoma of the head and neck to epidermal growth factor receptor inhibition. Mol Oncol. 2013; 7: 359-68.

25. Li H, Wawrose JS, Gooding WE, Garraway LA, Lui VW, Peyser ND, Grandis JR. Genomic analysis of head and neck squamous cell carcinoma cell lines and human tumors: a rational approach to preclinical model selection. Mol Cancer Res. 2014; 12: 571-82.

26. Parsons AB, Lopez A, Givoni IE, Williams DE, Gray CA, Porter J, Chua G, Sopko R, Brost RL, Ho CH, Wang J, Ketela T, Brenner C, et al. Exploring the mode-of-action of bioactive compounds by chemical-genetic profiling in yeast. Cell. 2006; 126: 611-25.

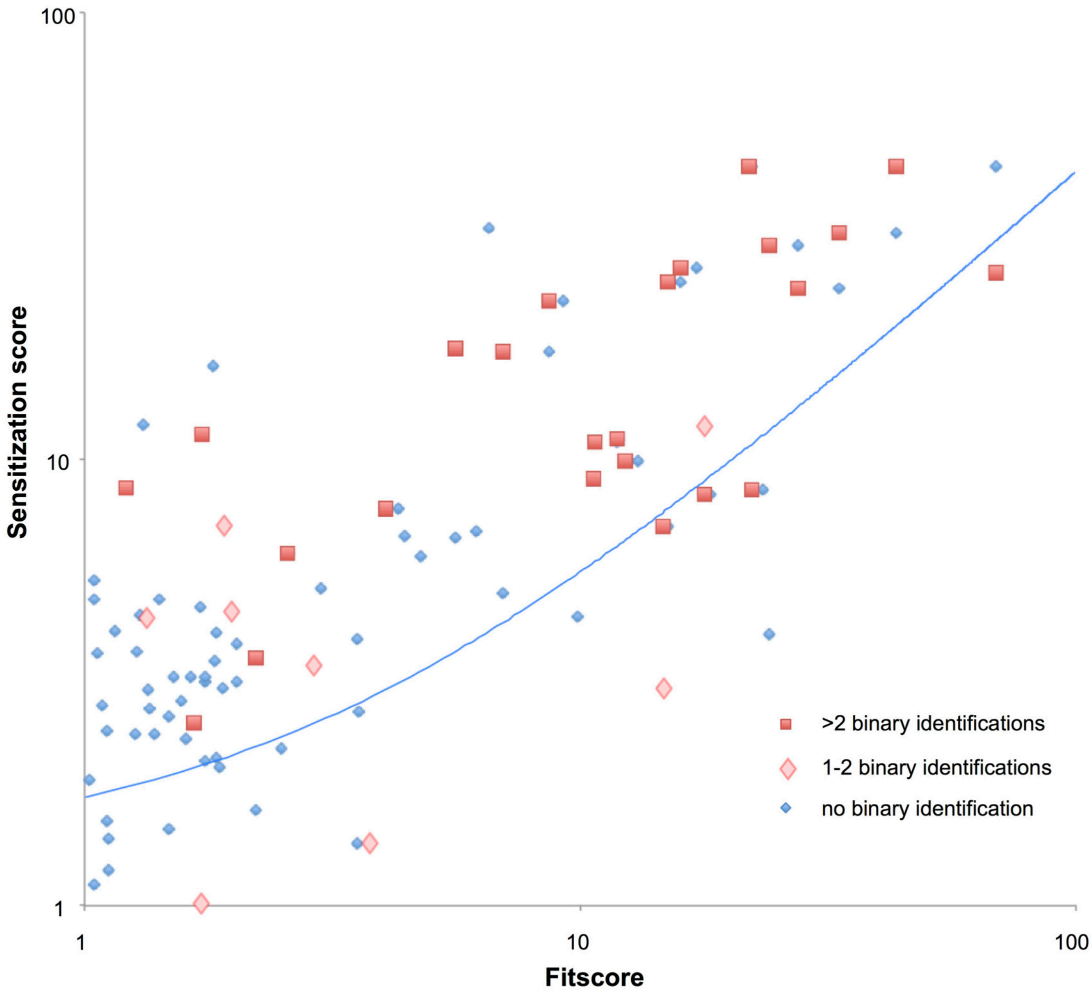

Supplementary Figure 1: Correlation of data from chemigenomics screens (fitscore, http://fitdb.stanford.edu; vs sensitivity score [26]) and cisplatin binary screens. Red square, genes nominated by 3 or more independent identifications in a binary screen; red diamond, genes identified in 1 or 2 screen; blue diamond, gene not identified in binary screens.

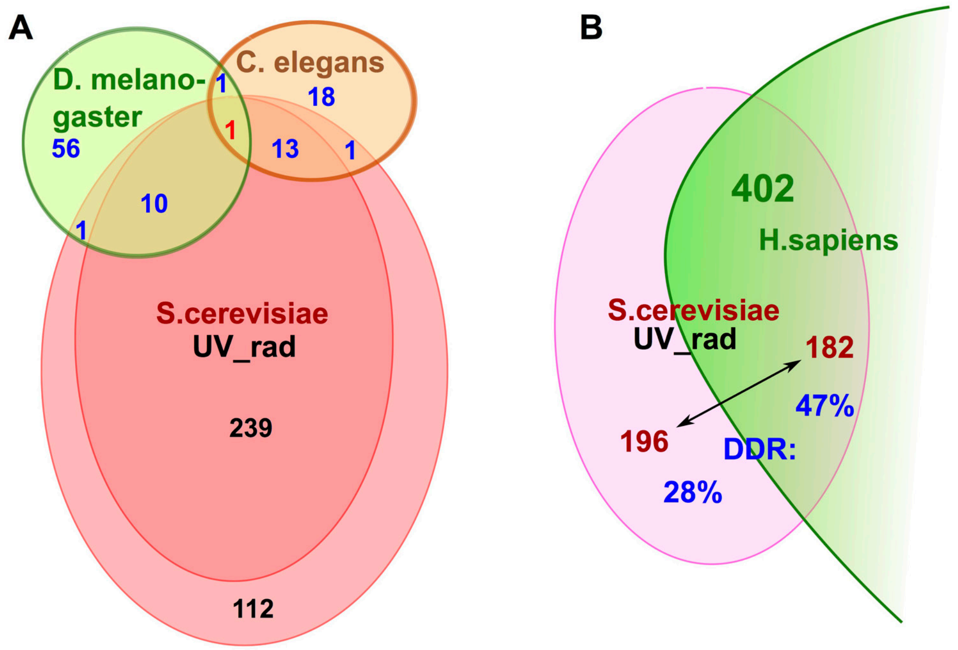

Supplementary Figure 2: Evolutionary conservation of *S. cerevisiae* UV and/or radiation resistance genes. **A.** Overlap between genes modulating sensitivity to UV and/or radiation in model organisms. *S. cerevisiae*: inner oval, number of genes from the UV_rad HTS≥2 & UV_rad LTS sets; outer oval, UV_rad HTS=1 found in the same chemogenomics clusters. The number of genes overlapping with those functionally identified in *D. melanogaster* or *C. elegans* screens is indicated graphically. **B.** Overall evolutionary conservation of *S. cerevisiae* UV_rad resistance genes in *H. sapiens*. Numbers shown in red font represent individual yeast genes, while green font is used for the number of human genes orthologous to their yeast counterparts. Fraction of the yeast genes annotated as involved in DDR is shown in blue.

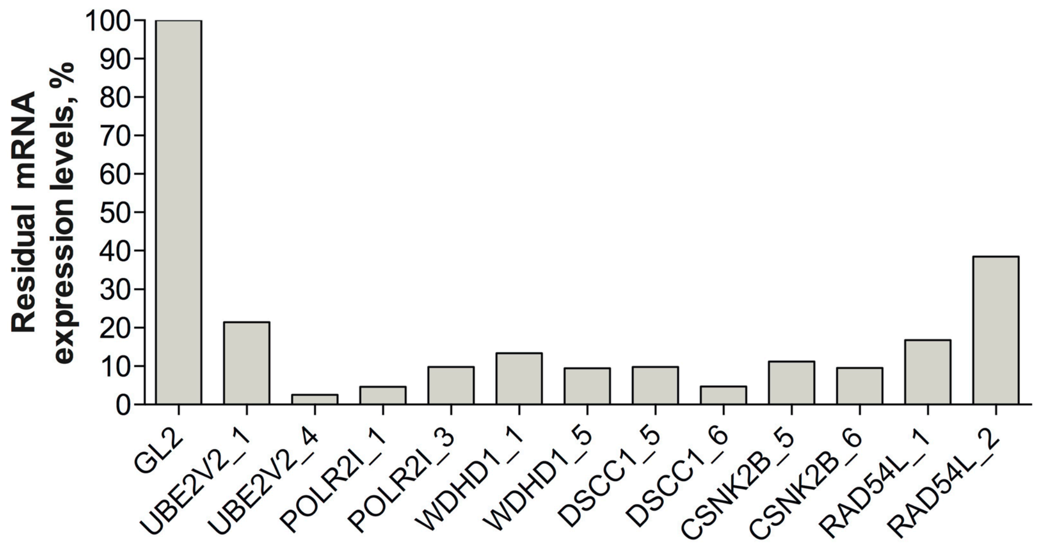

Supplementary Figure 3: Efficiency of target mRNA depletion by gene-targeting siRNAs. QRT-PCR was used to analyze mRNA depletion by indicated siRNAs 48 hours following depletion of SCC61 cells with siRNAs indicated to the genes indicated. Values are expressed in relation to transfection of cells with the negative control siRNA, GL2.

Supplementary Table 1: Candidate yeast genes regulating sensitivity to UV or ionizing radiation (UV_rad) nominated through low throughput screens (LTS) or high throughput screens (HTS), and functional clustering. Columns 1 and 2 provide Ensembl Gene ID and assigned gene name for each gene (GeneID; Gene Name). Columns 3-5 provide information on the nominating sources for each gene, including description in any LTS study (LTS pubs), or in at least two HTS publications (HTS pubs), including the number of times isolated; or the co-segregation of a candidate nominated by a single HTS study-nominated candidate in the functional clusters described in Supp Table 3 (in clusters). Column 6 and 7 note whether the gene has *D. melanogaster* (fly) or *C. elegans* (worm) orthologues annotated for UV_rad. Column 8 notes whether the gene has human homologues (human). Column 9 indicates whether the gene is known to function in cisplatin resistance in *S. cerevisiae (S.c. cisplatin; see also Supp. Table 2)*, and column 10 whether it is defined as functioning in DNA damage response (DDR) in *S. cerevisiae* (DDR in S.c).

See Supplementary File 1

Supplementary Table 2: Candidate yeast genes regulating sensitivity to cisplatin nominated through low throughput screens (LTS) or high throughput screens (HTS), and functional clustering. Columns 1 and 2 provide Ensembl Gene ID and assigned gene name for each gene (GeneID; Gene Name). Columns 3-5 provide information on the nominating sources for each gene, including description in any LTS or HTS study (binary pubs), or in at least two chemigenomics publications (CGS sets), including the number of times isolated; or the co-segregation of a candidate nominated by a single chemigenomics study in the functional clusters described in Supp Table 3 (in clusters). Column 6 notes whether the gene has human homologues (human). Column 7 (S.c. UV_rad) indicates whether the gene is known to function in resistance to UV or ionizing radiation in *S. cerevisiae (S.c. UV_rad; see also Supp. Table 1)*, and column 8 whether it is defined as functioning in DNA damage response in *S. cerevisiae* (DDR in S.c.).

See Supplementary File 2

Supplementary Table 3: Yeast clusters enriched for genes sensitizing to UV/Rad- and/or cisplatin. Columns 1-3 provide cluster numbers (Cluster), Ensembl Gene ID (GeneID) and assigned gene names (Gene Name). Filled boxes in Columns 4-10 provide information on the provenance, functionality, and homology to selected model organisms, as follows: Column 4 (UV_rad-H), gene identified as UV_rad HTS≥2 & UV_rad LTS; Column 5 (UV_rad-L), gene identified as UV_rad HTS=1; Column 6 (UV_rad-hom), functional orthologs in fly and/or worm; Column 7 (cispl-B), cisplatin resistance candidates found in binary screens; Column 8 (cispl-CG-H), cisplatin candidates found in at least two chemigenomics screens (high statistical support); Column 9, (cispl-CG-L), cisplatin candidates found in only one chemigenomics screen (gray shading: no statistically significantly enrichment in any screen) ; Column 9 (yeast DDR), gene annotated as involved in response to DNA damage; Column 10 (HS_hom), genes have unambiguous orthologs in humans.

See Supplementary File 3

Supplementary Table 4: Chemosensitivity profiles of clusters 5 and 7. Left column lists chemical compounds for which mutations in genes in clusters 5 or 7 cause increased sensitivity, versus the complete set of other genes profiled in chemosensitivity profiling [18]. Column 2, the number of drug concentrations at which each compound was screened. For each compound, the number of concentrations showing very significant (t-test <10E-7) or significant (t-text <10E-2) differences versus the complete set of profiled genes is indicated in columns 3 and 4 (for cluster 5), and 5 and 6 (for cluster 7). Mechanism of action for each compound is briefly summarized in column 7.

See Supplementary File 4

Supplementary Table 5: *D. melanogaster* genes involved in modulating sensitivity to UV and ionizing radiation. Left column, Ensembl Gene IDs for *Drosophila* genes annotated (see Supplementary methods for details) as regulating sensitivity to UV and ionizing radiation. Each gene is paired with (right column) homologous *S. cerevisiae* genes with (green highlight) or without (orange highlight) annotation for function in these processes or detection in screens for UV_rad sensitizing genes. *Drosophila* genes with relevant functions, but lacking a *S. cerevisiae* orthologue are also indicated (no highlight).

See Supplementary File 5

Supplementary Table 6: *C. elegans* genes involved in modulating sensitivity to UV and ionizing radiation. Ensembl Gene IDs for *C. elegans* genes annotated (see Supplementary Methods for details) as regulating sensitivity to UV and ionizing radiation, paired with homologous *S. cerevisiae* genes with (green highlight) or without (orange highlight) annotation for function in these processes or detection in screens for UV_rad sensitizing genes. *C. elegans* genes with relevant functions, but lacking a *S. cerevisiae* orthologue are also indicated (no highlight).

See Supplementary File 6

Supplementary Table 7: Human homologues of yeast, worm, and fly genes identified as modulating sensitivity to UV, ionizing radiation, and cisplatin. Human genes are listed by Ensembl Gene ID and EntrezGene ID. For each gene, orthologues with cisplatin or UV_Rad phenotypes conserved in *S. cerevisiae* (yeast), *D. melanogaster* (fly), or *C. elegans* (worm) is noted (blue and green highlight). Gene Ontology (GO) annotation of the human gene for functions in DNA damage response (DDR), cell cycle control, or chromatin remodeling is noted (violet highlight). Ranks reflect the confidence in assigning the human genes as implicated in modulating sensitivity to UV_rad and/or cisplatin, based on metrics described in Supplemental Methods, with 1 indicating highest confidence, and green highlight indicating the most validated gene set.

See Supplementary File 7

Supplementary Table 8: List of siRNAs from QIAGEN used to deplete the indicated genes for function-testing experiments

Gene Name	siRNAs
REV3L	Hs_REV3L_1
	Hs_REV3L_10
UBE2V2	Hs_UBE2V2_1
	Hs_UBE2V2_4
POLR2I	Hs_ POLR2I_1
	Hs_ POLR2I_3
WDHD1	Hs_ WDHD1_1
	Hs_ WDHD1_5
DSCC1	Hs_ DSCC1_5
	Hs_ DSCC1_6
CSNK2B	Hs_ CSNK2B_5
	Hs_ CSNK2B_6
RAD54L	Hs_ RAD54L_5
	Hs_ RAD54L_11

Supplementary Table 9: Specific primer pairs from Thermo Fisher Scientific used in quantitative RT-PCR experiments to measure mRNA levels for indicated genes following siRNA-mediated depletion

Gene Name	Assay ID
UBE2V2	Hs00163342_m1
POLR2I	Hs01042086_g1
WDHD1	Hs00173172_m1
DSCC1	Hs00225430_m1
CSNK2B	Hs00365835_m1
RAD54L	Hs00269177_m1
REV3L	Hs01076848_m1

The assays were validated with a 4-fold, 4-point dilution curve of cDNA for each gene assessed. Expression of genes was normalized to the housekeeping gene POLR2F. For POLR2F, primers used were, Forward: TGCCATGAAGGAACTCAAGG; Reverse: TCATAGCTCCCATCTGGCAG; Probe: 6fam-CCCCATCATCATTCGCCGTTACC-bhq1.
